# Reliability and validity of the Turkish version of the vestibular rehabilitation benefit questionnaire

**DOI:** 10.3906/sag-1904-59

**Published:** 2021-04-30

**Authors:** Bülent GÜNDÜZ, Yasemin APAYDIN, Arzu GÜÇLÜ-GÜNDÜZ, Burak KABİŞ, Hakan TUTAR

**Affiliations:** 1 Department of Audiology, Faculty of Health Sciences, Gazi University, Ankara Turkey; 2 Department of Physiotherapy and Rehabilitation, Faculty of Health Sciences, Gazi University, Ankara Turkey; 3 Department of Otorhinolaryngology, Faculty of Medicine, Gazi University, Ankara Turkey

**Keywords:** Dizziness, peripheral vestibular disorders, vestibular rehabilitation

## Abstract

**Background/aims:**

Vestibular rehabilitation has an important role in the reduction of symptoms and in the recovery of patients in peripheral vestibular pathologies. Objective and subjective vestibular assessment tools are needed to assess vestibular rehabilitation effectiveness. The aims of the study were to develop the Turkish version of the internationally used Vestibular Rehabilitation Benefit Questionnaire (VRBQ) measure and to demonstrate the reliability and validity properties of the Turkish version in patients with peripheral vestibular hypofunction (PVH).

**Materials and methods:**

110 patients with unilateral PVH were included. For the analysis of test-retest reliability, Turkish version of VRBQ developed by translation-back translation method was applied to patients on the day of admission and the day after admission. To assess validity, patients were also evaluated with the VRBQ, Dizziness Handicap Inventory (DHI), Vertigo Symptom Scale-Short Form (VSS-SF), Vertigo Dizziness Imbalance (VDI) Questionnaire.

**Results:**

The VRBQ showed moderate to excellent internal consistency in total score and subscales scores (VRBQ-total Cronbach’s α = 0.91; dizziness α = 0.81; anxiety α = 0.68; motion-provoked dizziness α = 0.89; aypmtoms α = 0.88; health-related quality of life α = 0.87). In the test-retest reliability of VRBQ-total score was excellent (ICC = 0.94). The dizziness, the anxiety, the motion-provoked dizziness, symptoms and the health-related quality of life domains’ ICC were found respectively 0.90, 0.89, 0.84, 0.90, and 0.92. The construct validity of the VRBQ was determined. The VRBQ total was correlated with all parameters (r: 0.308 to –0.699, P < 0.05). The highest positive correlation was found between VRBQ total and DHI-functional (r: 0.680). The highest negative correlation was found between VRBQ total and VDI-total (r: –0.699).

**Conclusion:**

The results suggest that the Turkish version of the VRBQ is reliable and valid for evaluating the vestibular rehabilitation results.

## 1. Introduction

Peripheral vestibular hypofunction (PVH) is a common problem and affects the quality of life of patients with vestibular pathology some limitations in their daily life activities. The degree of activity limitations by vestibular disorders is largely unknown due to the insufficient specialized measures. Vestibular patients’ involvement in daily life has become an important notion in health care and rehabilitation. In the treatment of vestibular disorders, there is a growing interest in vestibular rehabilitationpeople negativelyhave whichreduce theirquality of life which has become one of the main treatments for these patients. The use of vestibular rehabilitation may be limited by the extent and location of the damage to the vestibular system and the condition of the visual and proprioceptive system [1]. Brainstem function and cerebellar integration are important for the success of vestibular rehabilitation. Objective and subjective vestibular assessment tools are needed to learn functional boundaries of the vestibular system and assess vestibular rehabilitation effectiveness.

The validity of a document is related to how well it is measured. There are different aspects of validity that may be established either subjectively or empirically. To illustrate, content validity refers to the relevance and coverage of a questionnaire, which means all items should be relevant, and all relevant issues should be covered [2]. Additionally, reliability is also an important property of a questionnaire. A useful measure must be reliable both in terms of internal consistency and consistency over time. Patients’ self-perception of dizziness and level of independence are essential aspects to be considered in daily life activity and for the selection of therapeutic approaches in vestibular rehabilitation. A relevant and valid evaluation tool should be used in vestibular rehabilitation assessments. Currently, there are many subjective assessment methods which were originally designed to evaluate patients suffering from vestibular dysfunction, such as the Dizziness Handicap Inventory (DHI), the Vestibular Disorders Activities of Daily Living Scale (VADL), the Vertigo Handicap Questionnaire (VHQ), the Activities-specific Balance Confidence Scale (ABC Scale), and the Vestibular Activities and Participation (VAP) measure [3–7]. These questionnaires differ from each other in relation to their purposes as well as their content [8]. 

Morris et al. recently made a relatively big contribution in this field by publishing a 35-item Vestibular Rehabilitation Benefit Questionnaire (VRBQ) in 2008 [9]. The same authors suggested a shorter version of the VRBQ with 22 items with reliability and validity in 2009 [10]. VRBQ was originally developed in English and applied to the English speaking population. VRBQ was developed to measure the effectiveness of vestibular rehabilitation easily and effortlessly. VRBQ evaluates dizziness, anxiety, motion-provoked dizziness and quality of life subscales in the questionnaire. The monitoring of the development of patients after vestibular rehabilitation is important for the creation of new programs and patient follow-up. Comprehensive assessment scales are needed to evaluate the developmental stages of patients receiving vestibular rehabilitation. 

The purposes of this study were to develop the Turkish version of the internationally used VRBQ measure and to demonstrate the reliability and validity properties of the Turkish version in patients with PVH.

## 2. Materials and methods

Firstly, permission has been received from the author who developed the original English VRBQ. Additionally, the study was approved by the Gazi University Ethics Committee (Date: 01.06.2016, Number: 77082166-604.01.02-). Each participant gave written consent to participate.

### 2.1. Translation and cultural adaptation

For the translation and the cultural adaptation of the VRBQ, the procedures suggested by Guillemin et al. were used in the translation of this measure into Turkish [11]. Firstly, it was translated from original English version into Turkish by two native Turkish speakers. One of them was an udiologist, the other one was an English linguist. Two specialists who were informed about this study merged the translated measures, and this merged text was prepared as the measure. Secondly, this prepared translation was back-translated into English by a translator who had not studied on the first translation. Thirdly, the expressions whose original versions were achieved through back translation were adapted, and those that were not in compliance with the original version were processed until the original version was reached. The final measure that was translated into Turkish and controlled on 37 people initially constructed. The questionnaire was finally shaped by researchers and it was applied to the participants. After that, face validity of the questionnaire was tested and the Turkish version of the questionnaire started to be used for the study.

### 2.2. Participants

A total of PVH (including vestibular neuritis, vestibular schwannoma, Meniere’s disease, labyrinthitis and other peripheral vestibular disease) diagnosed patients from the Department of Otorhinolaryngology were included. A total of PVH diagnosed patients from the Department of Otorhinolaryngology were included. Videonystagmography, video head impulse test, vestibular evoked myogenic potential test, vestibular function tests after otologic and neurotological examination were used to diagnose. Inclusion criteria were being between 18 65 years old, having vertigo or dizziness in the subacute or chronic phase of the vestibular disease, having no additional diseases of a neurological, orthopedic, circulatory system or vision that could cause vertigo, dizziness or imbalance. Exclusion criteria were using a vestibular suppressants and participating in a vestibular rehabilitation program for the last one month. In order to answer the questionnaire, participants who had problems in cooperation or communication were not included in the study. All vestibular suppressing medications used by the patients were stopped one week before the study.

All the 110 patients filled in the VRBQ, DHI, Vertigo Symptom Scale-Short Form (VSS-SF) and Vertigo Dizziness Imbalance (VDI) Questionnaire. The VRBQ was performed to the patients at first visit and second visit (after 24-h).

### 2.3. Measurements

Vestibular Rehabilitation Benefit Questionnaire (VRBQ): The VRBQ measures the difference between the current state of the participant and a situation that is “normal” for the individual. The questionnaire assesses individual’s dizziness on a typical day in the last week. It consists of 22 items that are categorized into 2 subscales covering all of the main aspects of dizziness and its impact. One of them is symptoms subscale which has dizziness, anxiety, motion-provoked dizziness and the other is health-related quality of life (HRQoL) subscale. Each item has its own response scale and all scales consist of 7 - point verbal scales. The raw scores from the questionnaire are transformed into a percentage scale to facilitate interpretation. 0% indicates the ‘best’ score and no deficiency in “normal” condition. 100% deficiency means that it is far from “normal”. Deficit score greater than 0% means the presence of symptoms before the onset of dizziness, loss of function or a decrease in health-related quality of life [10].

Dizziness Handicap Inventory (DHI): DHI quantifies the impact of dizziness on daily life by measuring self-perceived handicap [3]. It consists of 25 questions in three domains: ine for functional, seven for physical, and nine for emotional. The highest total score is 100 and the lowest is 0. There are three levels of disability according to the total score from the scale: 030 points as mild handicap, 31N-60 points as moderate handicap, and 61100 points as severe handicap [12]. Evaluations were made using the Turkish version of the scale [13].

Vertigo Symptom Scale-Short Form (VSS-SF): The scale is used to assess the frequency of symptoms such as imbalance, somatic symptoms, autonomic symptoms, anxiety and panic [14]. It consists of two subscales: Vertigo/Balance subscale associated with vertigo and balance disorders and Autonomic/Anxiety subscale associated with autonomic disorders and anxiety symptoms. The Vertigo/Balance subscale consists of 8 questions (032 points) and the Autonomic/Anxiety subscale consists of 7 questions (028 points). The patients are asked to answer these questions about their dizziness in the last 1 month. High scores indicate an increased incidence of vertigo-related symptoms in patients [12,14,15]. The Turkish version of the scale was used [14].

Vertigo Dizziness Imbalance (VDI) Questionnaire: The questionnaire is used to measure the frequency of disability of patients with vertigo and dizziness, and to determine how these problems affect the quality of patients’ daily life. The scale consists of 36 questions and 2 subscales including symptoms (14 questions) and health related quality of life (HRQoL) (22 questions). Patients are asked to choose the answer that best suits their situation. The maximum score for the symptom scale is 70 and the HRQoL is 110. A high score indicates that the symptoms in the daily life of the patients are poor and the quality of life is good [1416]. Evaluations were made using the Turkish version of the scale [14].

### 2.4. Statistical nalysis

AStatistical analysis of the study was conducted using the program “Statistical Package for the Social Sciences” (SPSS) Version 22.0 (SPSS Inc. Chicago, II, USA). The psychometric properties of the VRBQ were evaluated in terms of reliability and validity. The reliability of the VRBQ was evaluated via test–retest and internal consistency methods. Testretest reliability was calculated using the Intraclass Correlation Coefficient (ICC) score while the Cronbach alpha value was computed as an estimate of the internal consistency. The validity of the questionnaire was examined through the analysis of the construct validity. For the construct validity of the questionnaire, the VRBQ total score and its subscales were correlated by Spearman’s correlation coefficient with the total scores of the DHI, VSS-SF, VDI questionnaire and their subscales.

## 3. Results

The study was completed with 110 PVH patients 74 of whom were female (67.3%) and 36 were male (32.7%). The average age 47.33 ± 12.18 years (1865). The mean duration of the diagnosis was 11 months (IQR: 436 months).

- –The VRBQ showed moderate to excellent internal consistency in total score and subscales scores (VRBQ total Cronbach’s α = 0.91; izziness α = 0.81; nxiety α = 0.68; motion-provoked dizziness α = 0.89; symptoms DAα = 0.88; HRQoL α = 0.87) (Table 1). No matter which item of the measure was ignored, it was found with the remaining items that the internal consistency coefficients (Cronbach’s alpha) relating to the reliability analysis conducted on these remaining items were above 0.80 except anxiety domain. One of the reliability indicators of the measure was evaluated with a corrected item/total correlation coefficient, and a value above 0.50 was considered to be significant. 

**Table 1:** Internal consistency of the VRBQ–Turkish and its subscales.

	Cronbach’s alpha (n = 110)
VRBQ-symptoms	
Dizziness	0.81
Anxiety	0.68
Motion-Provoked Dizziness	0.89
Symptoms-Total	0.88
VRBQ-health-related quality of life	0.87
VRBQ-total score	0.91

The test-retest reliability of VRBQ total score was excellent (ICC = 0.94; 95% confidence interval, 0.920.96).The izziness domain’s ICC was found 0.90 (95% confidence interval, 0.860.94), the - D- nxiety domain’s ICC = 0.89 (95% confidence interval, 0.850.92), the motion-provoked dizziness domain’s ICC = 0.84 (95% confidence interval, 0.760.89), the ymptoms total domain’s ICC = 0.90 (95% confidence interval, 0.860.93 and the HRQoL domain’s ICC was found 0.92 (95% confidence interval, 0.880.94) (Table 2). All correlations were significant at < 0.001. These results indicate the high repeatability of the measurement. 

**Table 2:** Test-retest reliability of the VRBQ-Turkish and its subscales.

	ICC	ICC (95% CI)	p
VRBQ-Symptoms			
Dizziness	0.90	0.86–0.94	0.000
Anxiety	0.89	0.85–0.92	0.000
Motion-provoked dizziness	0.84	0.76–0.89	0.000
Symptoms-Total	0.90	0.86–0.93	0.000
VRBQ-health-related quality of life	0.92	0.88–0.94	0.000
VRBQ-total score	0.94	0.92–0.96	0.000

The analysis of construct validity of the VRBQ was performed with 110 patients (74 female, 36 male; age: 18 to 65). Correlations were calculated for total and subscale scores of the four questionnaires. The correlation results calculated for validity are shown in Table 3.

**Table 3:** Correlations between the VRBQ–Turkish and other outcome measures.

	Vestibular Rehabilitation Benefit Questionnaire											
	Symptoms								Health-related quality of life		Total	
	Dizziness		Anxiety		Motion-provoked dizziness		Total					
	r	P	r	P	r	P	r	P	r	P	r	P
DHI												
Functional	0.553	< 0.001	0.257	0.007	0.575	< 0.001	0.582	< 0.001	0.653	< 0.001	0.680	< 0.001
Physical	0.477	< 0.001	0.209	0.028	0.546	< 0.001	0.515	< 0.001	0.474	< 0.001	0.537	< 0.001
Emotional	0.476	< 0.001	0.405	< 0.001	0.428	< 0.001	0.531	< 0.001	0.576	< 0.001	0.621	< 0.001
Total	0.557	< 0.001	0.325	0.001	0.551	< 0.001	0.595	< 0.001	0.618	< 0.001	0.671	< 0.001
VSS-Short Form												
Vertigo/Balance	0.648	< 0.001	0.259	0.006	0.512	< 0.001	0.590	< 0.001	0.543	< 0.001	0.639	< 0.001
Autonomic/Anxiety	0.263	0.006	0.534	< 0.001	0.259	0.006	0.404	< 0.001	0.200	0.036	0.308	0.001
Total	0.485	< 0.001	0.374	< 0.001	0.421	< 0.001	0.513	< 0.001	0.435	< 0.001	0.530	< 0.001
VDI Questionnaire												
Symptoms	–0.552	< 0.001	–0.241	0.011	–0.521	< 0.001	–0.543	< 0.001	–0.515	< 0.001	–0.584	< 0.001
HRQoL	–0.526	< 0.001	–0.437	< 0.001	–0.556	< 0.001	–0.653	< 0.001	–0.579	< 0.001	–0.673	< 0.001
Total	–0.569	< 0.001	–0.409	< 0.001	–0.584	< 0.001	–0.665	< 0.001	–0.612	< 0.001	–0.699	< 0.001

The VRBQ-Dizziness subscale was correlated with all scales and subscales (r: 0.263 to 0.648, A- - S- - p < 0.05). The highest positive correlation was found between VRBQ-izziness and VSS-SF vertigo/balance subscale (r: 0.648). The highest negative correlation was found between VRBQ-izziness and VDI-otal (r: 0.569).

The VRBQ-nxiety was correlated with all scales and subscales (r: 0.209 to 0.534, pDDT-A < 0.05). The highest positive correlation was found between VRBQ-nxiety and VSS-SF autonomic/anxiety subscale (r: 0.534). The highest negative correlation was found between VRBQ-nxiety and VDI-HRQoL (r: 0.437).

The VRBQ-motion-provoked dizziness subscale was correlated with all scales and subscales (r: 0.259 to 0.584, < 0.05). The highest positive correlation was found between VRBQ-motion-provoked dizziness and DHI-unctional (r: 0.575). The highest negative correlation was found between VRBQ-motion-provoked dizziness and VDI–pAA--pFotal (r: 0.584).

The VRBQ-ymptoms total was correlated with all parameters (r: 0.404 to 0.665, < 0.05). The highest positive correlation was found between VRBQ-ymptoms total and DHI-otal (r: 0.595). The highest negative correlation was found between VRBQ-Symptoms total and VDI-T-S-pSTotal (r: 0.665).

VRBQ-HRQoL was correlated with all scales and subscales (r: 0.200 to 0.653, < 0.05). The highest positive correlation was found between VRBQ-HRQoL and DHI-unctional (r: 0.653). The highest negative correlation was found between VRBQ–HRQoL and VDI-Total (r: 0.612).

The VRBQ total was correlated with all parameters (r: 0.308 to 0.699, T-pF-- < 0.05). The highest positive correlation was found between VRBQ total and DHI-Functional (r: 0.680). The highest negative correlation was found between VRBQ-Total and VDI-otal (r: 0.699).

## 4. Discussion

Although the development of scales for quality of life requires a lot of time, adaptation of these scales to different languages and cultures has become widespread, which contributes to a global standard. 

 In the related literature, it is clearly seen that there is not a single scale evaluating the effectiveness of vestibular rehabilitation in every aspect. Generally, the evaluation of treatment efficacy is provided by a number of evaluations, both before and after treatment. For this reason, Morris et al. have developed a 22-item VRBQ and aimed to devise a detailed and practical assessment tool [10]. It is also evident in the related literature that there is no study other than the original version of the VRBQ. Therefore, this study has been the first which has translated and adapted the original version into a new one. In the Turkish version of the VRBQ, participants generally interpreted it as easy to understand and easy to implement. None of the questions used in the questionnaire were extracted because there was no difficulty in understanding and implementing the items in the questionnaire.

The present study explored the psychometric properties of the VRBQ in patients with PVH. The results of the study indicate that the Turkish version of VRBQ has moderate to strong measurement properties. Therefore, we can say that the VRBQ is a reliable and valid measurement tool for research and practice in patients with PVH. 

In this study, the VRBQ total and subscales scores showed moderate to excellent internal consistency with similar results of Morris et al. [10]. The VRBQ is a multifactorial measure questionnaire and provides multifactorial assessment to see effects of treatment [10]. Considering the subscales in our study, internal consistency is the highest in the VRBQ-motion-provoked dizziness (α = 0.89). On the other hand, the highest internal consistency of subscales was obtained for VRBQ-HRQoL (α = 0.92) in the original study. We also found that the internal consistency of VRBQ-HRQoL was 0.87. We believe that this result is as valuable as the Morris et al. results, even if it is not our highest internal consistency result [10]. Considering the subscales in our study, internal consistency is the lowest in the VRBQ-nxiety (α = 0.68) and lowest consistency for VRBQ-nxiety (α = 0.74) in the original study. Our study is similar to the original study in this respect. However, in our study, internal consistency of VRBQ-AAotal score was obtained higher than the original study (α = 0.91 > 0.73). This result shows that there is no problem in using the questionnaire in Turkish language in terms of internal consisteTncy.

Morris et al. also preferred the 24-h period for the test-rest reliability because dizziness was a fluctuating process [10]. Therefore, a 24-h period was chosen for the test-retest reliability in this study. Results of this study demonstrate that the first and second measurements of the VRBQ and its subscales are consistent with each other. As a result, the VRBQ subscales and total scores show high test-retest reliability with 24-h period. The excellent test-retest reliability of the VRBQ subtests reveals that 24-h training is appropriate. According to the test-retest reliability, all correlations were significant in total score and subscales ( < 0.001). These results indicate the high reproducibility of the measurement. In our study, test-retest ratio of VRBQ-otal score was observed 94% while in the original study it was found 92%. Thus, it is obvious that the data obtained is very close in both studies. 

In the current study, correlations were calculated for total and subscale scores of the four questionnaires. Correlations were interpreted as follows: 0.6, strong; 0.400.59, moderate; 0.20ourourourourpT- 0.39, weak; < 0.20, no correlation [17]. In our study, VRBQ symptoms and HRQoL showed moderate to strong correlations with DHI, VSS-SF and VDI questionnaire on the overall assessment except that weak correlation between VRBQ-HRQoL and VSS-SF autonomic/anxiety was . For the construct validity of the VRBQ, Morris et al. found that DHI and its subscales were strongly correlated with VRBQ and its subscales [10]. In the current study, the results also support the results of Morris et al. [10].

In our study, VRBQ total score showed a strong and moderate correlation with total scores of DHI, VSS-SF and VDI questionnaires. This result was interpreted as a result of strengthening the clinical use of the Turkish version of the questionnaire. In addition, a significant parallel correlation was found between the VRBQ subscales and DHI total, VSS-SF total and VDI total scores, which indicates that subtests are complementary to the test in terms of clinical use.

It is generally aimed to reveal the effect of the disease on the quality of life with these developed scales. Therefore, it is important to have a subtest that measures direct quality of life within the VRBQ questionnaire. One of the subtests of the VDI survey is quality of life (VDI-HRQoL). In our study, a strong correlation was found between VRBQ-HRQoL and VDI-HRQoL. This result suggests that the Turkish version of the VRBQ survey will be an important tool for assessing quality of life. All of the VRBQ subtests showed moderate and strong correlation with VDI-HRQoL. This result confirms that the Turkish version of the VRBQ test can give clinicians insight into the quality of life. Morris et al. have used SF-36 for the construct validity of the health-related quality of life subscale of VRBQ, and eventually found relationships with all parameters, even if it is weak [10]. However, we have used VDI questionnaire instead of SF-36. SF-36 is generally used to assess general health status and is not disease-specific. VDI questionnaire is specific to vestibular diseases and the use of VDI questionnaire in this study was found to be more appropriate. 

The most important issue that should be assessed functionally in patients with dizziness is the functional limitations in the quality of life and daily life. In our study- obtained the VRBQ subscores showed the highest positive and negative correlations with the DHI-Functional and VDI-HRQoL subscales. This results allow us to reach the conclusion that VRBQ has stronger evaluations of daily life movements and quality of life.

In conclusion, this study shows that the Turkish version of the VRBQ is a valid and reliable measurement tool with strong psychometric properties in patients with PVH. The Turkish version of VRBQ is considered to be a suitable tool to control and monitor the rehabilitative status of the patient with vestibular rehabilitation. Additionally, the current study is the first version of VRBQ in another language. It is that the VRBQ is a facilitating tool to measure the quality of life of adults with peripheral vestibular disorders, to allow the planning of treatment, and to evaluate the results, and that it can be integrated into standard practices.

## Informed consent

The study was approved by the Gazi University Ethics Committee (Date: 01.06.2016, Number: 77082166-604.01.02-). Each participant gave written consent to participate.
